# Investigating the Acute and Chronic Effects of Cardiovascular Exercise on Brain-Derived Neurotrophic Factor in Early Subacute Stroke

**DOI:** 10.1177/15459683251342150

**Published:** 2025-06-03

**Authors:** Bernat De Las Heras, Lynden Rodrigues, Jacopo Cristini, Eric Yu, Ziv Gan-Or, Nathalie Arbour, Alexander Thiel, Ada Tang, Joyce Fung, Janice J. Eng, Marc Roig

**Affiliations:** 1 Memory and Motor Rehabilitation Laboratory (MEMORY-LAB), Jewish Rehabilitation Hospital, Laval, QC, Canada; 2 School of Physical and Occupational Therapy, McGill University, Montreal, QC, Canada; 3 Feil and Oberfeld Research Centre, Jewish Rehabilitation Hospital, Center for Interdisciplinary Research in Rehabilitation (CRIR), Laval, QC, Canada; 4 Department of Human Genetics, McGill University, Montreal, QC, Canada; 5 The Neuro (Montreal Neurological Institute-Hospital), Montreal, QC, Canada; 6 Department of Neurosciences, Université de Montréal, Centre de Recherche du Centre Hospitalier de l’Université de Montréal (CRCHUM), Montreal, QC, Canada; 7 Department of Neurology and Neurosurgery, Faculty of Medicine, McGill University, Montréal, QC, Canada; 8 School of Rehabilitation Science, Faculty of Health Sciences, McMaster University, ON, Canada; 9 Centre for Aging SMART at Vancouver Coastal Health and Department of Physical Therapy, University of British Columbia, BC, Canada

**Keywords:** stroke, cardiovascular exercise, BDNF, brain plasticity, recovery, biomarker

## Abstract

**Background:**

Following stroke, a growth-promoting response resulting in heightened neuroplasticity occurs during the early subacute stages of recovery, a period during which the brain may be more responsive to therapeutical interventions. Given its central role in regulating neuroplastic processes and brain repair in animal models, brain-derived neurotrophic factor (BDNF) has been targeted as a potential biomarker for stroke recovery in humans, with interventions upregulating BDNF holding therapeutical potential. Cardiovascular exercise (CE) has been recommended for stroke rehabilitation, partly due to its potential to induce neural adaptations, including upregulation of BDNF.

**Objectives:**

To examine the effects of CE on BDNF in individuals at early subacute stages of recovery.

**Methods:**

Seventy-six participants within 3 months of first-ever ischemic stroke were randomly assigned to 8 weeks of either CE plus standard care or standard care alone. To measure the chronic and acute responses to exercise in serum BDNF levels, blood samples were collected before and immediately after a graded exercise test conducted at baseline, 4, and 8 weeks. The potential role of the BDNF Val66Met polymorphism in modulating the BDNF response was also explored. Data were analyzed following an intention-to-treat approach.

**Results:**

Despite clinically important increases in cardiorespiratory fitness, CE did not induce significant chronic or acute changes in serum BDNF. Furthermore, the response to CE was not associated with changes in cardiorespiratory fitness and clinical outcomes or was modulated by Val66Met.

**Conclusions:**

These findings indicate that CE has a limited capacity to upregulate circulating BDNF in subacute stages of stroke recovery.

**Trial Registration:**

Exercise and Genotype in Sub-acute Stroke: https://clinicaltrials.gov/study/NCT05076747.

## Introduction

Following stroke, there is a time-limited window of heightened neuroplasticity and responsiveness to therapy during the initial weeks of recovery.^1^ In animal models, this critical period extends for 1 month, during which a growth-promoting phase fosters profound functional and structural adaptations in the brain underlying both spontaneous and treatment-induced recovery.^2^ These adaptations include multiple processes such as changes in gene expression, neural excitability, dendritic spine turnover, axonal sprouting, and remapping of neural networks, all regulated by growth-promoting molecules.^2^

In humans, this critical period of neural malleability, known as the early subacute period of recovery, is estimated to occur within the first week to 3 months post-stroke.^3^ During this early stage of recovery, wherein nearly all restoration from impairment tends to occur, motor rehabilitation interventions appear to induce significantly greater recovery gains compared to interventions initiated in later stages.^4^ Nevertheless, unlike in animal models, the specific neurobiological mechanisms underlying this critical period of recovery in individuals post-stroke remain poorly understood.

Brain-derived neurotrophic factor (BDNF), the most abundant neurotrophin in the brain, plays a crucial role in neural repair by binding to the tropomyosin receptor kinase B (TrkB) receptor and initiating intracellular signaling processes driving functional and structural neural change.^5^ Rodent studies of post-ischemic lesions have demonstrated the protective and restorative actions of BDNF, including mitigating cell death, facilitating synaptic plasticity, and improving functional recovery.^6^ BDNF has been shown to mediate early post-stroke motor recovery by regulating synaptic plasticity, underscoring its potential as a biomarker for stroke recovery.^6,7^

Genetic factors also play a significant role in stroke recovery by influencing neuroplasticity and neural repair mechanisms.^8^ Due to its capacity to interfere with the activity-dependent secretion of BDNF, the BDNF Val66Met polymorphism, a common variant of the BDNF gene involving a substitution of valine (Val) for methionine (Met) at codon 66, has been associated with suboptimal post-stroke recovery.^9^ Studying the BDNF Val66Met polymorphism and its impact on BDNF protein levels could enhance our understanding of the neurobiological processes underlying stroke recovery and the effect of interventions on this biomarker of neuroplasticity.

BDNF is an activity-dependent neurotrophic factor, with its expression, secretion, and action susceptible to interventions with the potential to enhance neural activity.^10^ Cardiovascular exercise (CE) is a simple yet effective intervention to protect and maintain brain function through its capacity to promote key neurobiological processes within the nervous system.^11^ In animal models, CE interventions have been shown to support functional recovery after stroke, in part by stimulating processes such as synaptic plasticity and upregulation of neurotrophic factors, with BDNF playing a crucial role in mediating CE-induced recovery after stroke.^12^

In neurotypical individuals, a single bout of CE (BDNF_acute_) transiently increases circulating BDNF concentrations.^13^ Although less consistently, BDNF increases have also been reported after chronic interventions involving multiple bouts of CE (BDNF_chronic_).^14^ Additionally, studies in animal studies have suggested amplified BDNF_acute_ responses following chronic CE programs, indicating an increased BDNF_acute_ responsiveness.^15^ Reduced BDNF levels, which have been observed in patients with stroke, have been associated with poorer long-term functional outcomes in some studies.^16^ Similar to neurotypical individuals, although with more inconsistent findings, CE interventions after stroke have shown to have the potential to modulate circulating BDNF levels, with increases reported following a single bout of CE and interventions involving multiple CE sessions.^17^

All existing studies, however, focused on patients at chronic stages of stroke recovery (>6 months),^18^ thereby neglecting the critical period during which the brain might be more responsive to the neuroplastic effects of CE.^1^ We conducted a study to evaluate the effects of CE on circulating BDNF levels in individuals <3 months of post-stroke recovery. Over an 8-week period, during which participants received either CE + standard care or standard care alone, BDNF_chronic_ levels were measured at rest and BDNF_acute_ after a single CE session. We also examined the associations between BDNF responses and changes in recovery outcomes, as well as the potential influence of Val66Met in the response. We hypothesized that CE would increase both BDNF_chronic_ and BDNF_acute_ response but that carrying the Val66Met would attenuate the response.

## Materials and Methods

### Design

**Figure 1.**
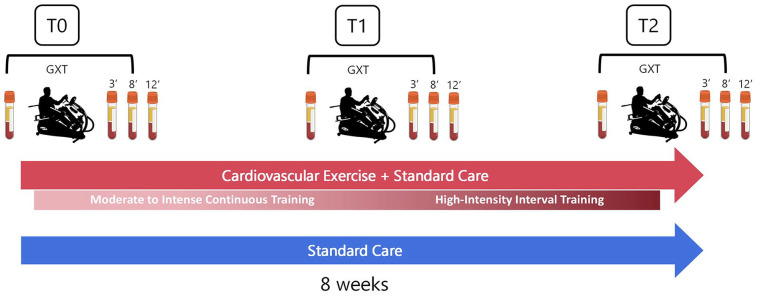
Study design with blood collection evaluations at baseline (*T*0), 4 weeks (*T*1), and 8 weeks (*T*2). Participants were randomly assigned to either an 8-week CE training in addition to standard care (red arrow) or standard care alone (blue arrow). The 8-week CE intervention consisted of 4 weeks of moderate to intense continuous training followed by 4 weeks of high-intensity interval CE training. To measure the effects of CE training on circulating BDNF_chronic_, blood samples were taken before the graded exercise test (GXT) at rest. To measure BDNF_acute_, blood samples were taken at 3, 8, and 8 minutes after the GXT. These measurements were conducted in both groups at each time point (*T*0 − *T*2). Abbreviations: CE, cardiovascular exercise; GXT, graded exercise test.

In this registered randomized controlled trial (NCT05076747) participants were assigned to either an 8-week CE training in addition to standard care or standard care alone (Figure 1). Given the unequal allelic frequency of the Val66Met in different populations,^19^ the randomization sequence allocated more participants in the CE training group to increase the power for detecting effects of this polymorphism on the BDNF response to CE. Assessments occurred at baseline (*T*0), 4 weeks (*T*1), and 8 weeks (*T*2). Each assessment consisted of 2 experimental sessions 48 hours apart that comprised the assessment of clinical motor outcomes and cardiorespiratory fitness with blood collection for BDNF. Information regarding participant’s characteristics and clinical information were collected at *T*0. Enrollment occurred between June 2018 and July 2023. The site ethics board approved the study (Centre de Recherche de Readaptation du Montréal, CRIR-1265-0817) and all participants provided written informed consent.

### Participants

We only included individuals with first-ever ischemic stroke within the early subacute stages of recovery (7 days-3 months). Participants had to be between 40 and 80 years old, present with no upper-limb musculoskeletal or neurological conditions other than stroke, have sufficient ability/capacity to perform the CE training and assessments safely, and have sufficient cognitive/communicative capacity to understand instructions. Individuals were excluded if they had a hemorrhagic stroke, cognitive impairment/dysphasia affecting informed consent, absolute contraindications to exercise,^20^ or were concurrently enrolled in another CE training program.

### Assessments

#### Baseline Assessments

At baseline (*T*0), stroke severity and cognitive status were assessed with the National Institutes of Health Stroke Scale (NIHSS)^21^ and the Montreal Cognitive Assessment (MoCA),^22^ respectively. The age-adjusted Charlson Comorbidity Index (CCI) was employed to assess pre-existing comorbidities.^23^ Self-reported physical activity levels were measured at each time point using the physical activity scale for people with disabilities (PASIPD).^24^ Participants were instructed not to engage in moderate- or high-intensity physical activity 24 hours before the assessments.

#### Cardiorespiratory Fitness

Measurement of peak oxygen uptake (VO_2_peak in mL/kg/minute) with a graded exercise test (GXT) is the gold standard for determining cardiorespiratory fitness. A symptom-limited GXT utilizing a protocol validated for individuals with stroke was performed on a whole-body recumbent stepper (NuStep T4r, Michigan, USA).^25^ During the GXT, heart rate (HR) was measured continuously while blood pressure (BP) and rate of perceived exertion (RPE) were recorded every 2 minutes. The GXT was also used to determine maximal HR (HR_max_, beats per minute [bpm]) and peak power output (PPO) expressed in Watts. Indications for test termination followed current guidelines.^26^

#### Clinical Motor Outcomes

Upper-limb motor impairment was evaluated using the Upper-Limb Fugl-Meyer Assessment (UL-FMA),^27^ while changes in upper-limb function on the affected side were assessed with the Box and Block Test (BBT).^28^

#### Blood Collection and Analysis

Blood collection was carried out by a nurse, with patients instructed to abstain from eating for at least 2 hours before the GXT. An antecubital intravenous line was placed in the non-paretic arm, with a waste sample collected before each blood extraction and the line flushed after each draw. A 5 mL blood sample was collected in a vacutainer serum separator tube 10 minutes before the GXT and at 3, 8, and 12 minutes post-GXT. BDNF_chronic_ was assessed from the sample collected at rest before the GXT, while BDNF_acute_ was calculated as the change between the sample collected at rest before the GXT and the average concentrations measured at samples 3, 8, and 12 minutes post-GXT (Figure 1).^29^

It was not possible to perform blood collection exactly at the same time of day (eg, 8 AM) for all participants; however, for each individual participant, samples were consistently collected at the same time of day across timepoints (*T*0, *T*1, and *T*2). Upon collection, blood samples were clotted for 1 hour, resting at room temperature, followed by 30 minutes at ~4°C, and then centrifuged at 2200*g* for 15 minutes. The resulting serum was then aliquoted into 250 μL cryovials and stored in a −80°C freezer. Identified as one of the best-performing assays,^30^ the Biosensis Mature BDNF Rapid™ enzyme-linked immunosorbent assay Kit was employed to determine BDNF concentrations.

#### Genotyping

Genomic DNA was extracted from red blood cells or saliva samples (DNA Genotek Inc., Canada) collected at baseline (*T*0), and genotyped using the Infinium Global Diversity Array-8 v1.0 from Illumina. DNA extraction and purification were processed by Genome Quebec (Quebec, Canada) using the QIAsymphony system (QIAGEN). Sixty-eight individuals were genotyped with sufficient DNA concentration for reliable genotyping (10 ng/µL). Standard quality control was performed using PLINK v1.9. Subjects were classified based on their genotype for the BDNF single nucleotide polymorphism rs6265 as homozygous for the Val allele (Val/Val), heterozygous (Val/Met), and homozygous for the Met allele (Met/Met) using PLINK v1.9 (Table 1). Individuals with Val/Met and Met/Met genotypes were combined to increase statistical power.^31^

**Table 1.** Baseline Demographic and Clinical Outcomes.

Outcome	CE + Standard care (n = 48)	Standard care (n = 28)
Age (years)	63.00 ± 11.39	65.35 ± 8.68
Sex (F/M)	11/37	10/18
BMI	27 ± 3.46	26.52 ± 4.01
Time since stroke (days)	68.12 ± 22.07	58.75 ± 24.03
Lesion location (%)		
Cortical	16	21
Cortico-subcortical	19	14
Subcortical	54	57
Cerebellar/Brainstem	10	7
NIHSS (0-42)	2.02 ± 2.20	1.92 ± 1.92
MoCA (0-30)	24.14 ± 4.85	23.21 ± 3.8
UL-FMA (0-66)	56.20 ± 10.24	59.14 ± 8.22
BBT_affected_ (blocks/min)	46.8 ± 13.25	48.10 ± 12.65
Cardiorespiratory fitness (VO_2_peak, mL/kg/min)	17.88 ± 5.49	18.12 ± 5.58
SNP rs6265		
Val/Val	27	18
Val/Met	13	7
Met/Met	3	0
CCI (age-adjusted)	4.57 ± 1.83	4.57 ± 1.66
Walking aid dependence (%)	15	13
Smoking history (%)		
Non-smoker	52	43
Former smoker	40	56
Current smoker	8	1
Medications (n)	5.07 ± 2.58	5 ± 2.22
Classification (%)		
AC	60	53
ACE	31	42
AP	46	60
BB	35	25
PSY	33	28
STA	79	100
Therapy sessions (n)		
Physiotherapy	8.87 ± 8.21	6.59 ± 5.78
Occupational therapy	11.5 ± 8.26	7.45 ± 5.98
Speech therapy	5.02 ± 8.81	2.15 ± 5.48
Δ*T*0 − *T*2 physical activity (METs h/d)	1.48 ± 5.39	−0.23 ± 5.28

Values are presented as mean ± SD.

Abbreviations: AC, anticoagulant; ACE, Angiotensin-Converting Enzyme; AP, antiplatelet; BB, beta-blocker; BBT, Box and Block Test; BMI, body mass index; CCI, Charlson Comorbidity Index; CE, cardiovascular exercise; F, female; M, male; Met, methionine; METs, metabolic equivalent of task; MoCA, Montreal Cognitive Assessment; NIHSS, National Institutes of Health Stroke Scale; PSY, psychoactive; SNP, single-nucleotide polymorphism; STA, statin; UL-FMA, upper-limb Fugl-Meyer; Val, Valine.

### Intervention

#### Cardiovascular Exercise

The CE + standard care group underwent a total of 24 CE training sessions over an 8-week period, with a frequency of 3 times a week and a 48-hour rest between sessions whenever possible. CE comprised 4 weeks of progressive moderate-to-vigorous intensity continuous training (MICT) followed by 4 weeks of progressive high-intensity interval training (HIIT), all conducted on a whole-body recumbent stepper ergometer (Figure 1). Each training session included 2.5 minutes of warm-up and cool-down at 35% of the PPO, along with the main training component at the targeted intensity. BP was measured at the beginning and the end of each CE session. To quantify the CE stimulus, HR, and Watts were continuously monitored during training via a pulse sensor (Polar H10, Kempele, Finland) and the stepper’s digital console, respectively. RPE (0-10) was assessed every 5 minutes throughout each training session with the modified Borg scale.^31^ Training variables, including the average percentage of maximal HR (%HR_max_), the average percentage of PPO (%PPO), total steps, and average RPE, were calculated for each session to quantify internal and external training workloads.

##### Moderate-to-Vigorous Continuous Training (Weeks 1-4)

MICT has been typically employed as a standard CE modality in stroke rehabilitation programs. Intensities were determined using the PPO associated with VO_2_peak during the GXT at *T*0 and progressively increased by 5% weekly from 65% to 80% PPO, to promote training adaptations. Session durations also increased from 20 to 35 minutes.

##### High-Intensity Interval Training (Weeks 5-8)

HIIT intensities were determined using the PPO corresponding to the VO_2_peak level achieved during the GXT at *T*1. The HIIT protocol comprised 8 × 60-second high-intensity intervals (8 minutes) interspersed with 7 × 60-second low-intensity intervals (7 minutes), totaling 20 minutes per session. This 60:60 interval ratio is optimal for sustaining high intensities.^33^ While high-intensity intervals began at 85% PPO and increased by 5% weekly until reaching 100% PPO, low-intensity intervals were kept constant at 35% PPO. To minimize sudden changes in BP while ensuring target intensities, the workload was progressively increased (15 seconds) before each high-intensity interval.

#### Standard Care Program

Standard care consisted of rehabilitation sessions conducted in the same center as the intervention and prescribed by the stroke clinical unit. In addition to routine health monitoring by physicians and nursing staff, standard care included physiotherapy, occupational therapy, and speech therapy sessions. The content, duration, and amount of rehabilitation varied among patients and were tailored to individual needs as determined by the stroke clinical unit, with each therapy session lasting 45 minutes. To examine potential differences between groups in standard care, we recorded the type and number of therapy sessions received by each patient from the beginning of the study to its conclusion.

### Statistical Analysis

Statistical analyses followed a prespecified plan with an intention-to-treat approach. Data were plotted using normality plots and histograms for inspection. The Shapiro–Wilk test was used to confirm normality for each variable. Baseline differences in participant characteristics and clinical variables between groups were assessed using *t*-tests or Wilcoxon tests. Linear mixed models were used to analyze differences in clinical motor outcomes (UL-FMA and BBT), cardiorespiratory fitness, and BDNF measures between groups across time points (*T*0 − *T*2). Each model included either BDNF_chronic_ or BDNF_acute_ as the dependent variable, with time point (*T*0 − *T*2), group, and their interaction as fixed effects. Covariates in the model included age, sex, and stroke severity (NIHSS). Body mass index was also entered into the model as a covariate due to its significant effect on BDNF levels.^34^ Participants were treated as a random effect to account for individual differences at baseline. Exploratory analyses combining data from both groups were conducted to measure BDNF_acute_ at baseline (*T*0). The Tukey’s Honest Significant Difference test was applied to identify statistically significant pairwise differences.

To examine the potential influence of Val66Met (Val/Val vs Val/Met + Met/Met), the allele variant was nested within the Time × Group interaction. Based on the Bayesian Information Criterion, log-likelihood ratio tests, and the data’s temporal dependence, an AutoRegressive (AR1) repeated measures covariance structure was deemed most appropriate. Assumptions for linear models, including normality in the distribution of random coefficients, were examined for all the variables in the model.

Standard least squares multivariate linear regression analyses were used to investigate associations between BDNF_chronic_ and BDNF_acute_ with changes in cardiorespiratory fitness and clinical motor outcomes. The same covariates—age, sex, stroke severity, and body mass index (BMI)—were included. Multicollinearity between predictor variables was assessed with the variance inflation factor with a threshold of ≤5, indicating unacceptable multicollinearity.^35^ All statistical analyses were performed with JMP (SAS Institute Inc, Cary, NC), version 17, and tested for significance at an alpha level *P* < .05.

## Results

**Figure 2.**
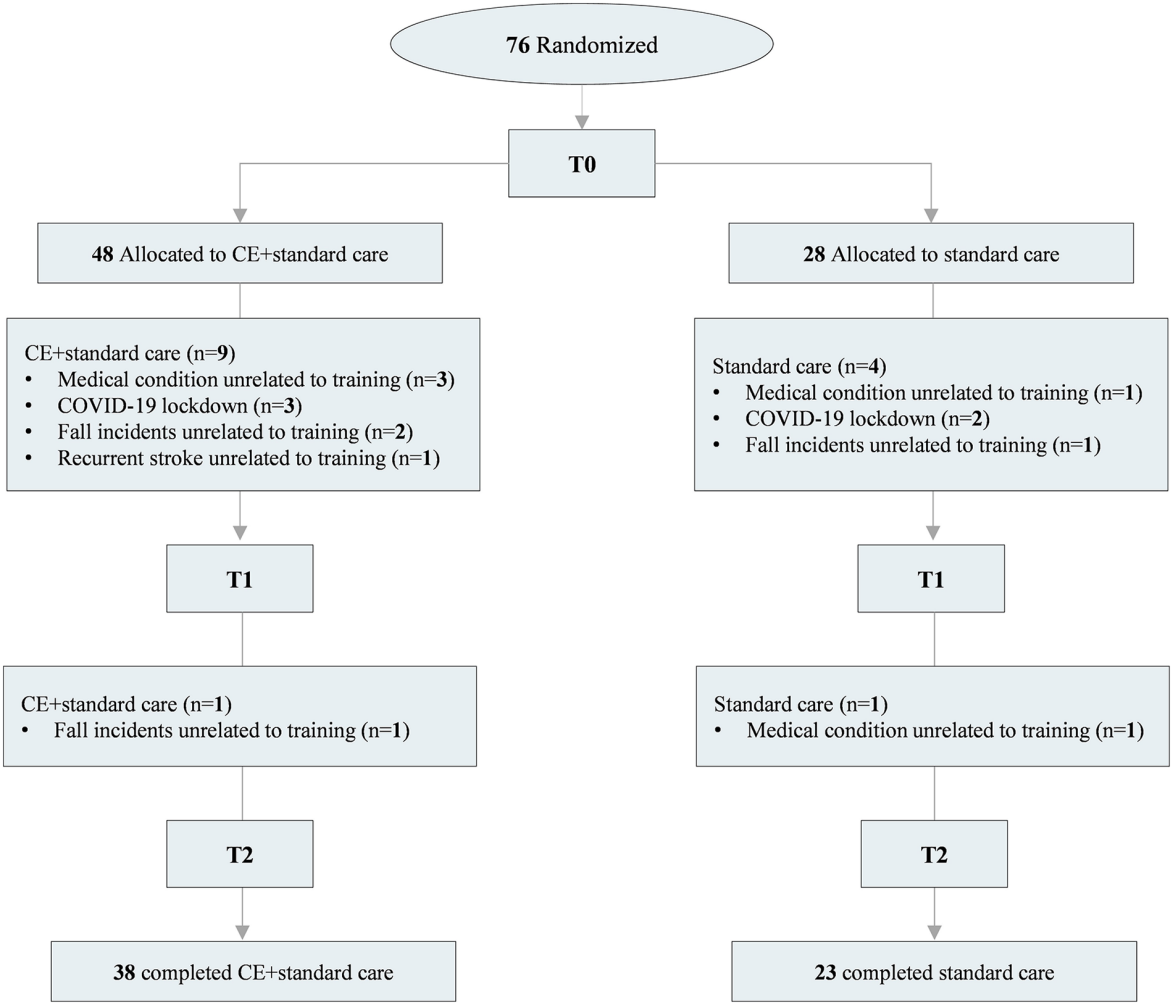
Consolidated Standards of Reporting Trials (CONSORT) flow chart of the randomized controlled trial. Abbreviations: CE, cardiovascular exercise; n, number of participants; *T*0, baseline; *T*1, 4 weeks; *T*2, 8 weeks.

Table 1 presents the participant’s characteristics and relevant clinical information for both groups at baseline. No serious exercise-related adverse events were reported during training. However, some participants dropped out from the study due to medical conditions unrelated to the intervention (n = 5), COVID-19 lockdown (n = 5), falls produced outside the training sessions (n = 4), and stroke recurrence (n = 1). The trial flow, including dropouts, is detailed in Figure 2. Data from all participants were included to measure BDNF_acute_ responses at *T*0, and an intention-to-treat approach was used for those who were assessed at least at *T*1.

On average, participants were 63.5 ± 10.2 years old (mean ± standard deviation) and initiated the study 65.1 ± 22.8 days after stroke. Most participants presented mild stroke severity, with an average NIHSS score of 2.01 ± 2.09, and an average MoCA score of 23.8 ± 4.48. No significant differences were observed at *T*0 between groups in terms of age, sex, BMI, time since stroke, lesion location, stroke severity, cognitive status, upper-limb impairment and function, pre-existing comorbidities (measured with age-adjusted CCI), walking aid dependence, smoking history, and the average number of prescribed medications. The amount of standard care provided during the participation in the trial and levels of physical activity outside of the trial were similar between groups. All participants assigned to the CE + standard care group who completed the study attended all 24 sessions. Internal and external training workloads during CE training are reported in Supplemental Table 1.

### Cardiorespiratory Fitness

No significant differences in cardiorespiratory fitness were observed between groups at baseline (*T*0). At *T*0, all participants obtained an average VO_2_peak of 18.43 ± 5.63 mL/kg/minute and attained a HR_max_ of 81 ± 13% of the age-predicted maximum and an average time to exhaustion of 10.49 ± 2.50 minutes during the GXT (Table 2). There was a significant effect of Time (*F*(2,78) = 16.76, *P* ≤ .0001), and a significant Time × Group interaction (*F*(2,78) = 13.46, *P* ≤ .0001) in cardiorespiratory fitness. While the standard care group showed no significant change in VO_2_peak from *T*0 to *T*2 (+0.27 mL/kg/minute, 95% confidence interval [CI] −2.19 to 1.64, *P* = .998), the CE + standard care group showed significant VO_2_peak improvements, with an increase of +2.76 mL/kg/minute (95% CI: 1.58 to 3.93, *P* < .0001) at *T*1 during MICT, further increasing by +1.64 mL/kg/minute (95% CI: 0.45 to 2.82, *P* < .0001) at *T*2 following HIIT, for a total gain of 4.43 mL/kg/minute from *T*0 (95% CI: 2.97 to 5.82, *P* < .0001).

**Table 2.** GXT Values at Baseline (*T*0), 4 Weeks (*T*1), and 8 Weeks (*T*2) for the CE + Standard Care and Standard Care Groups.

GXT value	*T*0	*T*1	*T*2
GXT (VO_2_peak, mL/kg/min)			
CE + Standard care	17.19 (0.82)	19.99 (0.84)	21.65 (0.85)
Standard care	18 (1.01)	18.11 (1.03)	18.34 (1.05)
GXT (%HR_max_)			
CE + Standard care	82.6 (1.87)	82.85 (2.05)	87.42 (2.0)
Standard care	78.89 (2.59)	80.73 (2.69)	81.66 (3.44)
GXT (minutes)			
CE + Standard care	11.23 (0.39)	13.02 (0.41)	13.97 (0.36)
Standard care	10.08 (0.55)	10.91 (0.47)	11.79 (0.64)

Measurements include maximum oxygen uptake (VO_2_peak), %HR_max_, calculated based on the HR relative to the age-predicted maximum and average time to exhaustion in minutes. Data are presented as least squares means with standard errors (SE).

Abbreviations: CE, cardiovascular exercise; GXT, graded exercise test; HR, heart rate; mL/kg/min, milliliters per kilogram per minute.

### Clinical Motor Outcomes

There was a significant effect of Time on upper-limb motor impairment and function using the UL-FMA (*F*(2, 99) = 15.61, *P* ≤ .0001) and BBT (*F*(2, 116) = 15.73, *P* ≤ .0001), with no significant Time × Group interaction for either measure (UL-FMA: *F*(2, 99) = 1.04, *P* = .355; BBT: *F*(2, 116) = 0.22, *P* = .801).

### BDNF Concentration

**Figure 3.**
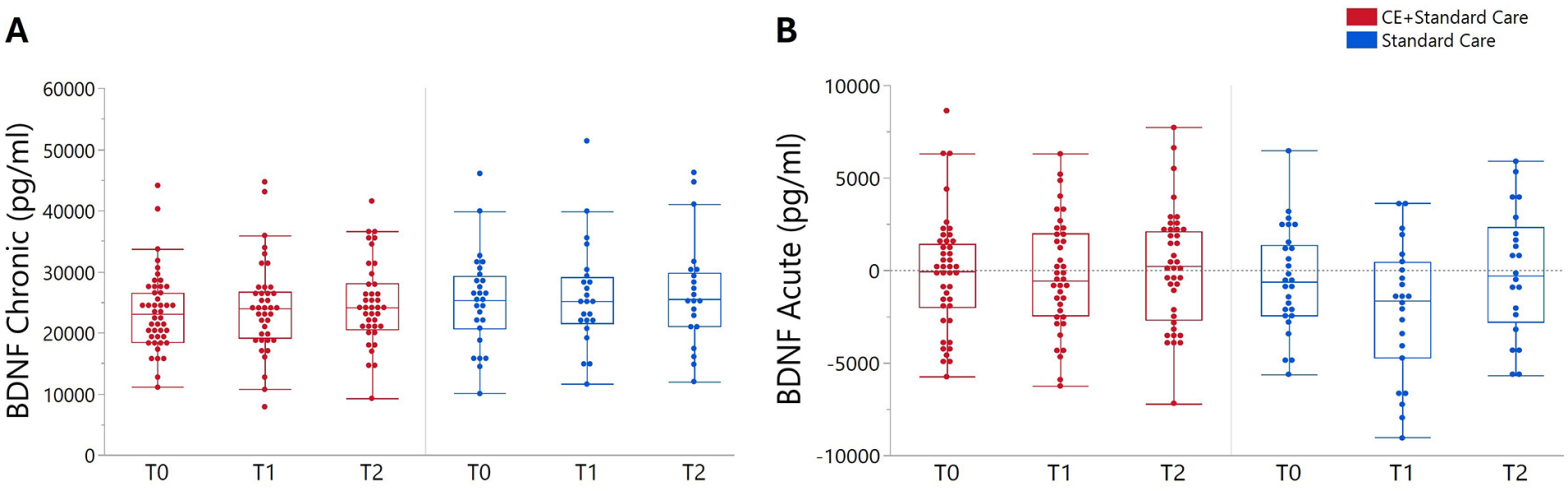
BDNF_chronic_ (A) and BDNF_acute_ (B) changes in BDNF concentration at baseline (*T*0), 4 weeks (*T*1), and 8 weeks (*T*2) following CE + standard care and standard care groups. Data are presented as raw values, with whisker plots representing interquartile range and potential outliers. Abbreviations: CE, cardiovascular exercise, pg/mL: picograms per milliliter.

Two participants did not go through blood sample collection, resulting in no BDNF data being available for analysis. BDNF_chronic_ and BDNF_acute_ changes for both CE + standard care and standard care groups are detailed in Table 3. At *T*0, no statistically significant differences in basal BDNF concentration were observed between groups (*P* = .275). Similarly, no significant effects of Time (*F*(2,186) = 1.08, *P* = .340) or Time × Group (*F*(2,186) = 0.06, *P* = .937) were identified for BDNF_chronic_ (Figure 3A).

**Table 3.** BDNF_chronic_ and BDNF_acute_ Concentration at Baseline (*T*0), 4 weeks (*T*1), and 8 weeks (*T*2) Following CE + Standard Care and Standard Care Groups.

Serum concentration (pg/mL)	*T*0	*T*1	*T*2
BDNF_chronic_			
CE + Standard care	23 260 ± 6433	24 084 ± 7554	24809 ± 6774
Standard care	25 094 ± 7682	25 946 ± 8586	26536 ± 8983
BDNF_acute_			
Δ CE + Standard care	233 ± 4060	−260 ± 3037	156 ± 3144
Δ Standard care	−507 ± 2821	−2173 ± 3622	−100 ± 3414

BDNF_chronic_ was assessed by comparing the basal concentrations at rest across the study time points, while BDNF_acute_ was determined as the difference between resting levels pre-GXT and the average concentration levels post-GXT (3, 8, and 12 minutes). Data are presented as mean and SD.

Abbreviations: CE, cardiovascular exercise; GXT, graded exercise test; pg/mL: picograms per milliliter.

No significant effects of Time (*F*(2,184) = 2.76, *P* = .065) or Time × Group (*F*(2,184) = 1.01, *P* = .364) were identified on BDNF_acute_ throughout the study (*T*0 − *T*2; Figure 3B). In the exploratory analysis combining both groups at *T*0 (n = 74) significant Time effects on BDNF_acute_ were observed (*F*(3,282) = 2.67, *P* = .047). Specifically, there was a non-significant increase from baseline to 3 minutes post-GXT (+574.93 pg/mL, 95% CI −672.18 to 1824.03, *P* = .632) and significant decrease between 3 and 12 minutes post-GXT (−1345.19 pg/mL, 95% CI −2593 to 96.47, *P* = .029).

Results showed no significant effects of Val66Met on either BDNF_chronic_ or BDNF_acute_ responses (Supplemental Table 2). Similarly, no significant associations were observed between BDNF_chronic_ and BDNF_acute_ responses and changes in clinical motor outcomes (UL-FMA and BBT) and cardiorespiratory fitness in either the CE + standard care or the standard care group (Supplemental Table 3).

## Discussion

Rehabilitative treatments capable of promoting neuroplasticity such as CE are believed to have therapeutic potential for stroke recovery, especially during the early post-injury, stages when the brain may be highly responsive to treatment.^1^ This study is the first to examine the effects of CE on BDNF levels in individuals with early subacute stroke. Contrary to our hypotheses, and despite clinically significant improvements in cardiorespiratory fitness (Table 2), 8-week progressive CE training did not significantly affect BDNF_chronic_ or BDNF_acute_ responses. Furthermore, BDNF responses were neither modulated by Val66Met nor associated with clinical motor outcomes.

CE is a core component of stroke rehabilitation with a well-established capacity to enhance cardiorespiratory health and metabolic function, reducing stroke recurrence risk factors, while also potentially supporting brain function and neural recovery.^12^ Studies on rodents have shown that several days of voluntary CE increase BDNF expression and its receptor TrkB in the brain, a molecular response mediating activity-dependent neuroplasticity processes supporting learning and memory, as well as neural repair post-stroke.^36^ However, the effects of CE on neuroplasticity and brain repair in individuals after stroke remain largely unknown, especially in the early stages of recovery.^18^

Despite the effectiveness of CE in improving cardiorespiratory fitness, our findings revealed no significant effects on BDNF_chronic_. The CE program led to significant increases in cardiorespiratory fitness, with average VO_2_peak increases of 4.43 ± 3.24 mL/kg/minute (+27.25%). These increases exceed the minimal clinical important difference of 3.0 mL/kg/minute,^37^ and surpass previously reported improvements in both subacute stroke individuals undergoing high-intensity CE interventions (1.46 mL/kg/minute)^38^ and chronic stroke populations in BDNF studies (Ploughman et al., 2019: 1.7 mL/kg/minute^39^; Hsu et al., 2021: 3.4 mL/kg/minute^40^). It is therefore unlikely that the lack of BDNF_chronic_ increase could be due to an insufficient exercise stimulus. This view was supported by our regression analysis, which found no significant association between changes in VO_2_peak and BDNF_chronic_ (Supplemental Table 3).

While unexpected, these results are consistent with the mixed evidence regarding the impact of CE training on BDNF_chronic_ in humans.^14^ In neurotypical populations, studies have presented conflicting findings, with some investigations reporting increased BDNF_chronic_ following long-term CE interventions, while others showing no change or even reductions.^14^ Only 2 studies have investigated the long-term effects of CE training on BDNF_chronic_ in patients with chronic stroke, with divergent results. One study (n = 23) reported significant increases after 12 weeks of HIIT,^40^ whereas another study (n = 52) reported no significant changes following 10 weeks of vigorous-intensity treadmill training compared to a group undergoing standard care.^39^

In contrast to other neurotrophins that are secreted constitutively, under resting conditions, BDNF remains within the cytoplasm and is only secreted in response to neural activity.^10^ This activity-dependent release is also evident following CE, where current evidence robustly supports BDNF_acute_ increases following a single CE session but does not consistently show BDNF_chronic_ after long-term training programs.^14^ This distinction could be of importance in stroke recovery, as the transient upregulation of intracellular signaling molecules in the brain like BDNF after a single exercise session initiates a biochemical cascade responsible for synaptic changes previously related to neural repair.^41,42^ Additionally, although long-term CE may not significantly increase BDNF_chronic_, animal studies suggest that long-term training programs can prime the BDNF_acute_ response to a single exercise session, indicating an adaptive mechanism.^15^

Our findings did not support a priming effect of CE training on BDNF_acute_ response, and the exploratory analysis combining both groups demonstrated only moderate effects on BDNF_acute_ in response to a GXT at *T*0. By using a GXT, we were able to measure BDNF_acute_ responses following a high-intensity CE session while also evaluating its association with cardiorespiratory fitness (VO_2_peak). These findings contrast with previous studies reporting BDNF_acute_ increases following a single vigorous CE session, including a GXT, in both neurotypical populations,^29^ and individuals in the chronic stage of stroke.^43^ Additionally, while studies in neurotypical populations show that several weeks of CE training can improve the BDNF response to a single bout of exercise (BDNF_acute_),^44^ our findings align with the only stroke study examining acute responses to training, which found no effect of 10 weeks of vigorous-intensity treadmill training on BDNF_acute_ immediately after a GXT.^39^

One possible contributor to the limited effects of CE on BDNF could be the stress and inflammatory processes characterizing early post-stroke stages. A stroke triggers a cascade of stress-related hormones (eg, corticosterone, cortisol) and pro-inflammatory molecules (eg, interleukin-6, tumor necrosis factor-alpha, or C-reactive Protein), which can persist during acute and subacute stages,^45,46^ attenuating BDNF mRNA levels and BDNF expression.^47,48^ Furthermore, while long-term CE has shown to offer both anti-inflammatory and stress-reducing benefits,^49^ animal studies show that a single CE session, when implemented at higher intensities, can stimulate pro-inflammatory responses and cause up to a 20-fold increase in corticosterone levels, thereby reducing BDNF expression.^41,50^ Given the high intensities attained during the GXT (Table 2), it is possible that our acute intervention could have acted as a stressor, potentially suppressing any BDNF_acute_ responses in early subacute stages, when growth-inhibiting processes are significant.^1,51^ These findings are consistent with previous animal work showing that a high-intensity motorized running session implemented 2 weeks post-stroke resulted in an attenuated BDNF response alongside significantly elevated serum corticosterone levels.^52^

While the inability of CE to promote BDNF, particularly BDNF_acute_ increases, could be related to the inhibitory processes characteristic of the early stages of post-stroke recovery, the fact that negative findings have also been reported in chronic stages post-stroke,^39^ suggests that other factors may also contribute. Taken together, our results suggest a high interindividual variability in the BDNF response to CE and the need of very large studies to capture significant effects. It is also important to note that several methodological and biological factors, such as sex, age, BMI, diurnal variations, fasting state, and, importantly, medications like anti-platelets commonly prescribed after stroke, have also been shown to significantly affect circulating BDNF levels.^34,53^ Although we made efforts to control for these variables, their influence on our findings cannot be ruled out entirely. Addressing some of these factors, although logistically complex, will be crucial for reducing the significant variability in BDNF measurements.

In line with previous evidence showing that peripherally measured BDNF cannot consistently predict recovery post-stroke,^54^ we found no significant associations between BDNF responses and changes in clinical motor outcomes in either CE + standard care or standard care groups (Supplemental Table 3). The lack of associations could be due to many reasons such as the null increase in BDNF in response to CE training, the fact that this intervention had little effect on clinical motor outcomes and, related to the latter, that most of our patients had relatively low impairment levels and thus a very limited room for functional improvement. These results contrast with pre-clinical evidence demonstrating that BDNF is crucial in mediating the positive effects that CE has on functional stroke recovery.^36^

One possible explanation for the discrepancies between animal and human studies could be the different sources from which BDNF is typically measured across species. In animal models, BDNF can be measured directly in the brain, whereas in humans, it is measured peripherally, assuming its concentration reflects central neural processes. Previous studies suggested that BDNF can be transported unidirectionally from peripheral circulation to the brain by crossing the blood–brain barrier (BBB)^55^ and that the brain might be the primary source of circulating BDNF both at rest and during CE.^56^ This view was supported by studies showing correlations between peripheral BDNF levels and central brain concentrations.^57^ However, this has been challenged by evidence indicating that neurotrophins, including BDNF, do not cross the BBB in large amounts unless they are conjugated with a molecular “Trojan horse.”^58^ When conjugated to a chimeric peptide, intravenous administration of BDNF reduces stroke volume and improves functional outcomes in rats with middle cerebral artery occlusion.^59^

This disparity between BDNF sources has also been observed during early post-stroke stages in animal models, where BDNF concentrations increase in the brain, while no changes are reported peripherally.^60^ This discrepancy underscores the need for caution in interpreting human studies and highlights the necessity for further studies to elucidate the role of circulating BDNF in central neural processes and its association with stroke recovery. BDNF is expressed primarily in the brain but it may also be found in blood platelets and vascular endothelial cells and it can be released by organs such as the heart, kidneys, liver, spleen and skeletal muscles, some of which increase their metabolic activity during exercise (see for review^61^). The exact origin of BDNF measured peripherally, especially in response to exercise, is still under debate.^29^ Employing techniques such as positron emission tomography could provide more sensitive measures of brain BDNF utilization through the TrkB/BDNF system.^62^

It has been hypothesized that individuals with 1 or 2 copies of the met allele of the Val66Met polymorphism may show a decreased response to neuroplasticity-based interventions due to diminished BDNF secretion.^63^ This study is the first to investigate the impact of the Val66Met polymorphism on serum BDNF levels in response to CE in individuals with stroke. In contrast to animal studies that consistently show the Val66Met polymorphism altering intracellular trafficking and activity-dependent BDNF expression, including in response to CE,^64^ BDNF_chronic_ and BDNF_acute_ were not influenced by Val66Met. Our findings align with other studies in neurotypical individuals reporting inconclusive results on the association between this genetic variant and BDNF levels following CE interventions,^31,65,66^ as well as its impact on post-stroke recovery outcomes.^67,68^ Nevertheless, these findings should be interpreted with much caution due to the larger sample sizes typically required to detect true effects in genetic studies,^69^ and the inherent variability of the BDNF response to exercise^29^ as well as of clinical stroke research.^70^

Finally, it is important to acknowledge that BDNF is not the only mediator of stroke recovery that can potentially be upregulated by CE. Cytokines such as IL-6 and other growth factors such as IGF-1 and VEGF have shown to be upregulated with CE and play an active role in neuroplasticity processes involved in brain recovery (see for review^18^). Larger studies will be needed to determine the impact of CE on the activity of these molecules in early stages post-stroke and their role on different aspects of recovery.

## Conclusion

This study is the largest trial investigating the effects of CE training on BDNF levels in individuals recovering from a stroke and the first trial exploring the effects in early subacute stages. Given that animal evidence suggests a period of heightened neuroplasticity and responsiveness to training during early stages of recovery,^1^ we expected a significant effect of CE on enhancing BDNF levels in early subacute stroke patients. Unexpectedly, our findings showed that, despite clinically significant improvements in cardiorespiratory fitness that by themselves justify the use of CE in early stages of stroke recovery, this intervention had a small effect on both BDNF_chronic_ and BDNF_acute_. Similarly, BDNF responses were not associated with changes in cardiorespiratory fitness, recovery outcomes or were influenced by BDNF Val66Met polymorphism, although the latter should be interpreted with caution due to the limited power of the study to detect potential effects of genotype. Factors such as high intersubject variability in the response, inflammation and stress responses during the early stages of post-stroke recovery, along with other methodological and biological factors, may have influenced these findings.

## Supplemental Material

sj-docx-1-nnr-10.1177_15459683251342150 – Supplemental material for Investigating the Acute and Chronic Effects of Cardiovascular Exercise on Brain-Derived Neurotrophic Factor in Early Subacute Stroke Supplemental material, sj-docx-1-nnr-10.1177_15459683251342150 for Investigating the Acute and Chronic Effects of Cardiovascular Exercise on Brain-Derived Neurotrophic Factor in Early Subacute Stroke by Bernat De Las Heras, Lynden Rodrigues, Jacopo Cristini, Eric Yu, Ziv Gan-Or, Nathalie Arbour, Alexander Thiel, Ada Tang, Joyce Fung, Janice J. Eng and Marc Roig in Neurorehabilitation and Neural Repair
